# 14–3-3ε: a protein with complex physiology function but promising therapeutic potential in cancer

**DOI:** 10.1186/s12964-023-01420-w

**Published:** 2024-01-26

**Authors:** Yue Zhang, Man Yan, Yongjun Yu, Jiangping Wang, Yuqi Jiao, Minying Zheng, Shiwu Zhang

**Affiliations:** 1 Tianjin University of Traditional Chinese Medicine, Tianjin, 301617 People’s Republic of China; 2 Department of Colorectal Surgery, Tianjin Union Medical Center, Tianjin, 300121 People’s Republic of China; 3 Department of Pathology, Tianjin Union Medical Center, Tianjin, 300071 People’s Republic of China

**Keywords:** 14–3-3ε, Cell cycle, Cell immunity, Invasion and metastasis, Chemoradiotherapy resistance

## Abstract

Over the past decade, the role of the 14–3-3 protein has received increasing interest. Seven subtypes of 14–3-3 proteins exhibit high homology; however, each subtype maintains its specificity. The 14–3-3ε protein is involved in various physiological processes, including signal transduction, cell proliferation, apoptosis, autophagy, cell cycle regulation, repolarization of cardiac action, cardiac development, intracellular electrolyte homeostasis, neurodevelopment, and innate immunity. It also plays a significant role in the development and progression of various diseases, such as cardiovascular diseases, inflammatory diseases, neurodegenerative disorders, and cancer. These immense and various involvements of 14–3-3ε in diverse processes makes it a promising target for drug development. Although extensive research has been conducted on 14–3-3 dimers, studies on 14–3-3 monomers are limited. This review aimed to provide an overview of recent reports on the molecular mechanisms involved in the regulation of binding partners by 14–3-3ε, focusing on issues that could help advance the frontiers of this field.

Video Abstract

**Supplementary Information:**

The online version contains supplementary material available at 10.1186/s12964-023-01420-w.

## Introduction

The 14–3-3 protein was first identified in bovine brain homogenate by Moore in 1967 [1], and its name is derived from the number of fragments in DEAE-cellulose chromatography and position of migration in starch gel electrophoresis [2]. 14–3-3 is a family of highly homologous proteins encoded by different genes and comprised different isoforms, which vary in number depending on the species. There are seven main isoforms with a molecular weight of 30 KDa in mammals, which are α/β, γ, ε, η, σ, θ/τ, and ζ. All isoforms share approximately 44% sequence homology; their protein structures are well conserved [3], and both homo-and heterodimers can be formed [4, 5]. 14–3-3 is abundant in the brain [6] (13.3 pg/mg soluble protein) and has also been detected in almost all tissues [7, 8]. Although 14–3-3 is mainly a cytoplasm protein, it is also found in the plasma membrane [9], nucleus, Golgi apparatus, chloroplasts, and mitochondria [10]. Notably, 14–3-3 binds to the phosphoserine/phosphothreonine motif in a sequence-specific manner [11]. 14–3-3 protein–protein interactions exhibit typical phosphorylation dependence, and three conserved binding motifs have been identified: RXXpS/TXP, RX (F/Y) XpS/TXP, and pS/TX COOH [12]. More than 1,200 binding partners of 14–3-3 have been identified, further demonstrating the important regulatory role of 14–3-3 in various processes, such as signaling, cell cycle regulation, neural development, and stress response [13, 14].

14–3-3ε, a member of the 14–3-3 protein family, is encoded by the *YWHAE* gene. In humans, *YWHAE* is located on the 17^th^ chromosome and involved in cell adhesion, cell cycle regulation, signal transduction, and other biological processes. It is closely associated with the development of numerous diseases, including cardiovascular and neurodegenerative diseases (e.g., Alzheimer’s disease, Parkinson’s disease, and Miller-Dieker syndrome). Interestingly, 14–3-3ε is associated with aggressive migration and poor prognosis in various cancers. Here, we reviewed the characteristics and functional roles of 14–3-3ε.

### Structure of 14–3-3ε

14–3-3ε is present in both monomeric and dimeric forms. The seven 14–3-3 isoforms are highly conserved in mammals, and each monomer consists of nine antiparallel α-helices (H1-H9) forming an amphipathic groove that can bind to (mainly phosphorylated) protein partners [15]. Additionally, the homologs of 14–3-3 are very similar in structure. The largest sequence variation between the subunits is found in the C-terminal extension; however, this extension is disordered [15]. This flexible region interacts with the positively charged phospho-binding pocket of 14–3-3.

The 14–3-3 proteins exist mainly as dimers, which form cup-shaped particles with two-fold symmetry and have central channels formed by helices H3, H5, H7, and H9. The central channel contains two ligand-binding grooves. The residues that form the inner surface of the dimer, including the two ligand-binding grooves, are highly conserved, whereas those forming the outer surface of the central channel exhibit a high degree of variability [15]. The 14–3-3 dimer interface in homodimers consists of helices H1 and H2 of one monomer interacting with helices H3 and H4 of the other monomer via a salt bridge and several other hydrophobic and polar contacts [16]. The 14–3-3 subtypes, which are highly conserved, exhibit significant differences in their tendency to form homodimers or heterodimers. These differences arise from minor yet structurally significant variations in their sequences [17]. Yang et al. investigated the formation of heterodimers among different 14–3-3 isoforms and found that the ε isoform displayed a higher affinity for binding with β, γ, and τ, resulting in virtually no detected homodimers [18]. Chaudhri et al. observed that the ε isoform tended to form heterodimers rather than homodimers [19]. All human 14–3-3 subtypes have a dimerization interface around the aperture, forming a conserved salt bridge between Arg-19 and Glu-92. In addition, Asp-21 and Lys-85 are also conserved in all human isoforms except in ε (ε residues are Glu-22 and Met-88). In the homodimeric structures, no side chains at these positions interact with each other. While 14–3-3ε acts as a subunit of the heterodimer, additional opportunities for hydrogen bonding will be created, which may account for its tendency to form heterodimers [18].

### Functions of 14–3-3ε

#### 14–3-3ε promotes cell proliferation by binding to calmodulin and apoptosis repressor with a caspase recruitment domain (ARC)

Increasing evidence shows that 14–3-3ε is important for cell proliferation processes. Luk et al. first identified a tight interaction between calmodulin and the 14–3-3ε helix α4 peptide, adding to the evidence on the role of 14–3-3ε in cell proliferation [20]. In vitro experiments also revealed that after serum stimulation, 14–3-3ε and calmodulin, together with Ca^2+^/CaM-dependent protein kinase II, were localized to the centrosome and spindle apparatus during interphase [20, 21]. This, in turn, promoted the cell proliferation process. Yes-associated protein (YAP), a nuclear effector of the Hippo signaling pathway, induces gene expression by activating DNA-binding transcription factors that control cell survival and proliferation [22, 23]. YAP was observed to bind to the 14–3-3 protein blocked nuclear translocation, which eventually inactivated YAP function and retained YAP in the cytoplasm [24]. Furthermore, ARC is highly expressed in the heart and protects the heart [25, 26]. Recent studies also showed that ARC increased the nuclear localization of YAP by binding to 14–3-3ε, which in turn promoted cell proliferation [27]. However, the exact binding site between ARC and 14–3-3ε requires further investigation.

#### 14–3-3ε promotes cell apoptosis by binding to cytochrome c (Cyt c), E2F, peroxisome proliferator-activated receptor (PPAR) and NMDA receptors

Apoptosis is a meticulously controlled programmed cell death mechanism that plays a vital role in the development and maintenance of multicellular organisms. Using the modified yeast two-hybrid system, 14–3-3ε was identified as a caspase-3 substrate [28]. 14–3-3ε mutant proteins cleaved by caspase-3 decrease the binding ability with Bad. Subsequently, Bad relocates to the mitochondria, where it interacts with Bcl-x(L) and facilitates the release of Cyt c and ultimately results in cell death [29]. Cyt c is central to the intrinsic apoptotic pathway activation and activates the cysteine cascade through its interaction with apoptotic protease activating factor-1 (Apaf-1). Cyt c also binds to the groove and convex surface of 14–3-3ε [28]. Thus, the interaction among Cyt c, 14–3-3ε, and Apaf-1 reveals a strict apoptotic regulatory network. The E2F family of transcription factors is an important regulator involved in apoptosis [30]. The E2F family consists of at least eight E2Fs and two DP subunits, of which the NLS region of DP-3 contains the 14–3-3ε binding site. Moreover, interaction between DP-3 and 14–3-3ε alters the apoptotic properties of E2Fs. In addition, 14–3-3ε is found in the promoter regions of some E2F target genes, and reduced 14–3-3ε levels induce apoptosis [31]. On the other hand, PPARs are ligand-activated receptors of the nuclear hormone receptor family. Three PPAR isoforms are essential for protection against apoptosis induced by oxidative and metabolic stress [32, 33]. When intracellular 14–3-3 levels are insufficient to control apoptosis, PPARα, γ, or δ, activated by their respective ligands, forms a heterodimer with RXR that binds to the PPRE site on the 14–3-3ε promoter and upregulates 14–3-3ε transcription. Upregulated 14–3-3ε enhances Bad binding and sequestration and reduces Bad interference with Bcl-2 and Bcl-x(L) protection [34–37].

The low-affinity neurotrophin receptor, also known as p75NTR, transmits signals that lead to neuronal cell death [38]. A protein called the p75NTR-associated cell death executor (NADE) interacts with the structural domain of p75NTR located inside the cell [39]. Using the yeast two-hybrid method, 14–3-3ε, NADE, and p75NTR form protein complexes to promote neuronal apoptosis in vivo [40]. Moreover, neuronal development is significantly affected by NMDA receptors [41], which depend on the presence of NR1 and NR2C to properly function. Overactivation of NMDA receptors leads to apoptosis of striatal neurons. In a trial by Chen et al., NR2C phosphorylation by PKB to regulate the trafficking of NMDA receptors was achieved by promoting the binding of 14–3-3ε proteins to NR1/NR2C heteromeric receptors [42].

#### 14–3-3ε regulates cellular autophagy by crotonylation and binding to chloride intracellular channel 4 (CLIC4)

Autophagy is a process through which eukaryotic cells use lysosomes to degrade cytoplasmic proteins and damaged organelles under the regulation of autophagy-related genes [43]. Bioinformatics analysis has revealed that 14–3-3 proteins dominate the leucine-mediated crotonylome [44]. Crotonylation increases the instability of the molecule and disrupts the 14–3-3ε amphiphilic pocket, preventing the interaction of 14–3-3ε with its binding partners. Leucine deprivation-induced crotonylation of 14–3-3ε results in the release of protein phosphatase, Mg ^2+^/Mn^2+^-dependent, 1B (PPM1B) from the 14–3-3ε interaction, and active PPM1B dephosphorylates Unc-51-like kinase 1, which triggers autophagy [45]. CLIC4, a member of the chloride intracellular channel family, is a polymorphic protein that regulates various biological functions [46, 47], and mass spectrometry (MS) results have shown its interaction with 14–3-3ε [48]. In addition, siRNA-induced inhibition of CLIC4 prevents the interaction of CLIC4 with 14–3-3ε, leading to an increase in beclin-1 [49]. This suggests that CLIC4 plays an important role in autophagy, and this process requires 14–3-3ε involvement.

#### 14–3-3ε regulates cell differentiation and migration

The 14–3-3ε and ζ proteins accelerate the turnover of δ-catenin by interacting with phosphorylated δ-catenin in neural progenitor cells. Simultaneously, they facilitate the formation of F-actin via the catenin/Ro-GTPase/Limk1/cofilin signaling pathway, which regulates neuronal differentiation [50]. In the nervous system, Rho GTPases can adjust the shape of dendrites in differentiated neurons. In this context, 14–3-3η and 14–3-3ε were identifies as binding partners of p190RhoGEF, indicating the potential role of the 14–3-3 proteins in neural morphogenesis [51].

Semaphorin affects axon growth and regulates cell migration, thus playing an important role in the development of the central nervous system [52]. As a receptor of semaphorin, plexin is considered an important player in central nervous system development [53]. Plexins are Ras/Rap family GTPase-activating proteins (GAPs), and the GAP activity of plexin is controlled by protein kinase A (PKA), which phosphorylates plexin and generates binding sites for 14–3-3ε [54]. Furthermore, these PKA-mediated 14–3-3ε–plexin interactions block the interaction between plexin and its Ras GAP substrate, Ras2, thus acting as an antagonist of semaphorin signaling [55]. 14–3-3ε is also required for normal neuronal migration in vivo, and YWHAE-deficient mice exhibit defects in brain development and neuronal migration. Specifically, 14–3-3ε interacts with phosphorylated NUDEL, which in turn interacts with LIS1 to form a complex that regulates centrosomal protein location, ultimately leading to neuronal migration [56, 57].

#### 14–3-3ε regulates the cell cycle by binding to cell division cyclin 25 (CDC25), dynein and the human ether-a-go-go related gene (*hERG*) channel

CDC25 expression is tightly regulated throughout the cell cycle [58, 59]. In the cell division process, CDC25 plays a crucial role in activating the cyclin-dependent kinase 1 (CDK1)–cyclin B complex by dephosphorylating CDK1 [60]. This complex enhances the phosphatase activity of CDC25C by phosphorylating it, creating an irreversible auto-amplification loop that drives cells into mitosis. During interphase, CDC25C binds to 14–3-3, leading to CDC25C sequestration in the cytoplasm and transient dissociation of 14–3-3ε, followed by dephosphorylation of Ser-287, eventually leading to nuclear translocation of CDC25 [61]. Uchida et al. suggested that the binding of 14–3-3ε to CDC25C inactivated NLS, allowing the N-terminal NES of the CDC25C protein to predominate [62]. Meng et al. proposed two models for the regulation of CDC25C function by 14–3-3ɛ and 14–3-3γ. The first model suggested that both 14–3-3ɛ and 14–3-3γ recognized CDC25C through unique F/V pockets, with 14–3-3ɛ and 14–3-3γ forming primary and secondary contacts with CDC25C, respectively. The second model suggested that 14–3-3ɛ specifically recognized CDC25C through the F/V pocket, whereas 14–3-3γ was free to interact with other molecules [63]. In addition, 14–3-3ε and 14–3-3γ formed a complex with Centrin2 that affected the cell cycle by regulating centrosome replication by inhibiting CDC25C function [64]. Another study demonstrated that 14–3-3 binding to CDC25B played a crucial role in regulating the redistribution of CDC25B from the nucleus to the cytoplasm [65]. The interaction between 14–3-3ε and PKA-phosphorylated CDC25B-S321 leads to the localization of CDC25B in the cytoplasm and regulates the G2/M transition during mitotic progression in fertilized mouse eggs [66].

Dynein is the main motor responsible for the movement and force toward the negative end of microtubules, which plays a key role during interphase and cytokinesis [67]; it consists of the heavy, light, intermediate, and light intermediate chains [68]. Partner of Inscuteable (Pins) is a modular protein that coordinates the activity of dynamin and kinesin-73 in the cortex of animal cells to orient the spindle. Specifically, this process involves the 14–3-3ζ/ε heterodimer binding to the dynein adaptor NudE to complete the dynein connection [69]. The Khc73 stalk/14–3-3/NudE pathway establishes a physical connection that enables the coordination of multiple motor proteins, resulting in the precise positioning of the spindle. The LIC2-dynein complex is another major cortical protein complex that ensures spindle orientation [70, 71]. LIC2-dynein achieves spindle orientation through its ability to anchor astral microtubules in the cortex via its interaction with cortical 14–3-3ε and 14–3-3ζ [71].

hERG K^+^ channels are important for human cardiac action potential repolarization [72, 73] and signaling pathways that lead to cell proliferation [74]. β1-adrenaline stimulation regulates hERG K^+^ channels through interaction with multiple pathways, including direct binding to the cyclic adenosine monophosphate pathway [75] and phosphorylation by PKA [76]. Meanwhile, 14–3-3ε is the most abundant 14–3-3 isoform in the heart. The binding of 14–3-3 occurs simultaneously at the N- and C-terminals of the hERG channel, stabilizing the longevity of the PKA phosphorylation state of the channel and leading to prolonged effects of adrenergic stimulation on hERG activity [77, 78].

#### 14–3-3ε maintenance of intracellular electrolyte homeostasis by binding to Na^+^, K^+^-ATPase, cofilin, Nedd4-2 and NCX

Na^+^/K^+^-ATPases (sodium pumps) are prevalent in mammalian cell membranes and play a key role in maintaining intracellular electrolyte homeostasis [79]. 14–3-3ε binds to the N-terminal structural domain of Na^+^/K^+^-ATPase, and this binding is dependent on PKC phosphorylation [80]. In the presence of arrestin and spinophilin, the binding of 14–3-3ε to Na^+^, K^+^-ATPase is completely blocked, demonstrating that arrestin and spinophilin might regulate trafficking by inhibiting the binding of 14–3-3ε [81].

The epithelial sodium channel (ENaC), also known as amiloride-sensitive sodium channel, is an ion channel that is selectively permeable to sodium ions. ENaCs are located in the parietal membrane of polarized epithelial cells, particularly in the renal collecting ducts. ENaC promotes Na^+^ reabsorption across the apical membrane of epithelial cells in the distal renal unit, thereby maintaining extracellular volume, sodium homeostasis, and blood pressure [82]. Insulin increases α-ENaC expression and function in mouse cortical collecting duct cells, and cofilin is involved in the regulation of α-ENaC by interacting with 14–3-3ε, β, or γ [83]. Suppression of 14–3-3ε expression significantly attenuates aldosterone-stimulated ENaC expression and Na^+^ transport and increases the interaction between Nedd4-2 and ENaC, similar to the effect of 14–3-3β knockdown [84, 85]. This shows that 14–3-3β and 14–3-3ε, as obligatory heterodimers, interact with Nedd4-2, blocking its interaction with ENaC and resulting in increased apical ENaC density and Na^+^ transport [85]. Shen et al. reported that insulin-induced selective binding of 14–3-3ε to Forkhead box protein o1 (FoxO1) stabilized FoxO1 in its phosphorylated form, thereby increasing apical ENaC density and Na^+^ transport [86].

Na^+^/Ca^2+^ exchanger (NCX) is an antiporter membrane protein that removes Ca^2+^ from cells. There are three NCX isomers, namely, NCX1, NCX2, and NCX3, and the interaction of NCX2 with 14–3-3ε has been confirmed using a yeast two-hybrid approach. Further studies revealed that 14–3-3ε also inhibited NCX1 and NCX3 activity and that the phosphorylated form of NCX had stronger binding affinity [87]. Plasma membrane Ca^2+^-ATPase (PMCA) is an ATP-driven Ca^2+^ pump critical for maintaining low resting cytoplasmic Ca^2+^ levels in eukaryotic cells. In He-La cells, 14–3-3ε binds to PMCA4 [88]. Linde et al. investigated the relationship between 14–3-3ε and PMCA and found that 14–3-3ε co-immunoprecipitated with PMCA3 and PMCA1, which was subsequently verified by a glutathione S-transferase assay [89].

#### 14–3-3 regulates cell immunity through activation of T cells and regulation of the retinoic acid-inducible gene I (RIG-I) translocator

The 14–3-3 protein family plays integral roles in viral infections and innate immunity. The SH2 domain-containing leukocyte protein of 76 kD (SLP-76) consists of 533 amino acid residues involved in the regulation of T cell receptor-related signaling pathways [90]. The binding of SLP-76 phosphorylation at serine 376 to 14–3-3ε and ζ results in a negative signal to regulate T cell activation [91]. RIG-I is a cytosolic pathogen recognition receptor that binds to pathogen-associated molecular pattern motifs within viral RNA during acute infection, thereby mediating immune signaling [92]. After the recognition of viral RNA, antiviral signaling requires redistribution of RIG-I from the cytosol to the membrane, where it binds to the adaptor mitochondrial antiviral signaling protein (MAVS). 14–3-3ε is an RIG-I binding protein that forms a “RIG-I transposon” with RIG-I, 14–3-3ε, and tripartite motif-containing protein 25 for MAVS interactions and immune signaling in acute RNA viral infection [93].

### 14–3-3ε exerts its physiological functions via multiple signaling pathways

14–3-3ε is involved in several signaling pathways to regulate normal physiological functions of the body, including tumor growth factor (TGF) β-mediated signaling, Wnt/β-catenin pathway, PI3K pathway, NF-kB signaling, and Hedgehog (Hh) signaling.

#### 14–3-3ε enhances TGFβ-mediated signaling by interacting with type II TGFβ receptors (TβRII) and eukaryotic initiation factor-2α (EIF2α)

**Fig. 1:**
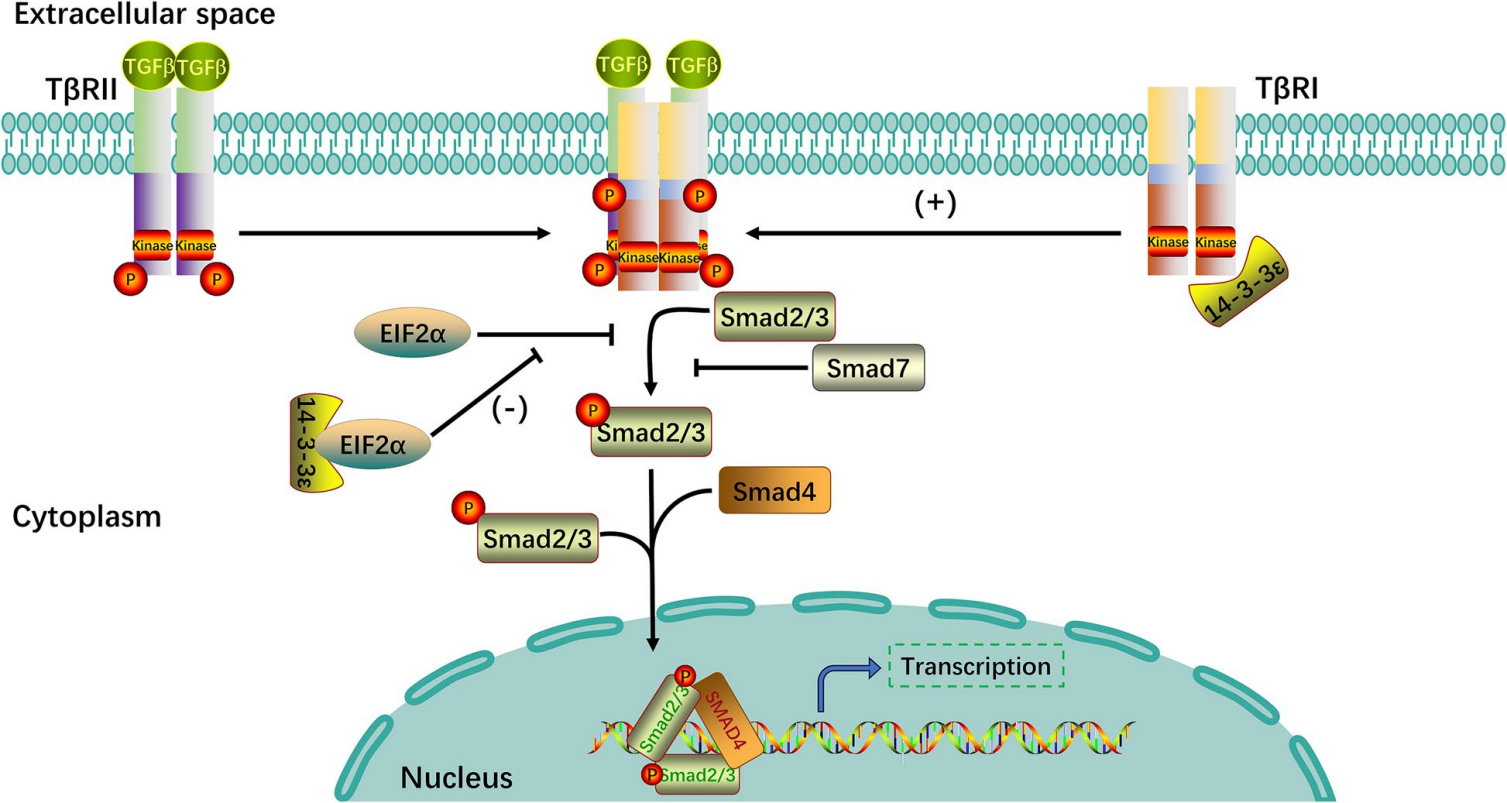
Roles of 14–3-3ε in TGFβ-mediated signaling. 14–3-3ε enhances the TGFβ signaling pathway in two ways. First, 14–3-3ε interacts with TβRI to enhance the TGFβ signaling pathway. Second, co-expression of 14–3-3ε with EIF2α counteracts the inhibitory effect of EIF2α on TGFβ-mediated signaling. TGF, tumor growth factor; TβRI, type I TGFβ receptor; EIF2α, eukaryotic initiation factor-2α

The TGFβ signaling pathway is a highly differentiated, versatile, and efficient cell signaling network that is involved in a diverse set of cellular processes including cell proliferation, differentiation, apoptosis, embryonic development, tissue homeostasis, inflammatory response, and immune regulation [94]. The TGFβ family is categorized into two distinct pathways based on the variations in signaling mediators and effectors: the canonical (Smad-dependent) and noncanonical (Smad-independent) pathways [95]. The mammalian genome encodes eight Smad proteins, including five receptor-activated Smads (R-Smads; Smad1, 2, 3, 5, and 8), one common Smad (Smad4), and two repressive Smad proteins (I-Smads; Smad6 and 7). TGFβ receptors can be classified into type I TGFβ receptors (TβRI), TβRII, and type III TGFβ receptors based on their structural and functional properties. TβRI and TβRII are single transmembrane serine/threonine kinase receptors with intrinsic kinase activities that are required for the mediation of TGFβ signaling. TβRII initially phosphorylates the intracellular region of TβRI, and the phosphorylated TβRI then recruits and phosphorylates R-Smads at their C-terminal serine residues [94]. Activated R-Smads are released from type I receptors and usually form heterotrimers comprising two R-Smad molecules and one Smad4 molecule. The complex then translocates to the nucleus to regulate the target gene expression [94, 96]. 14–3-3ε interacts with TβRI and activates the TGFβ signaling pathway [97]. Further, EIF2α can also play an inhibitory role in TGFβ receptor signaling, and co-expression of 14–3-3ε with EIF2α offsets the inhibitory effect of EIF2α on TGFβ-mediated signaling [98]. A schematic of this process is shown in Fig. 1.

#### 14–3-3ε regulates cell proliferation through the Wnt/β-catenin and PI3K pathways

**Fig. 2:**
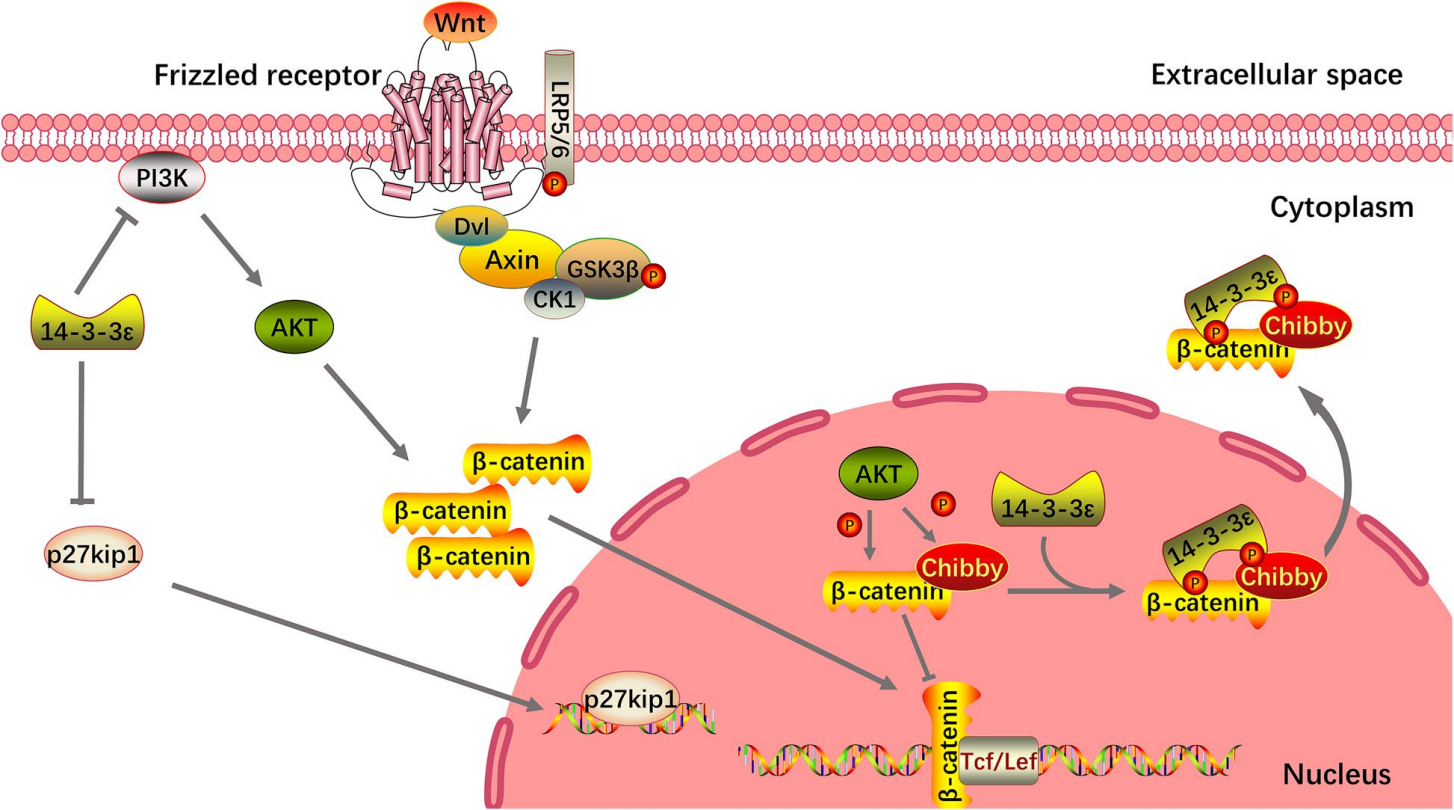
14–3-3ε regulates cell proliferation through the Wnt/β-catenin and PI3K pathways. 14–3-3ε inhibits PI3K relocalization to the plasma membrane and thus inhibits PI3K/AKT/β-catenin signaling. Chibby, 14–3-3ε, and β-catenin in the nucleus form a complex that leads to β-catenin entry into the cytoplasm, which antagonizes β-catenin signaling. Meanwhile, 14–3-3ε promotes cell proliferation by inhibiting p27kip1 expression

CTNNB1 encodes for β-catenin, and its accumulation in the nucleus is indicative of active Wingless (Wnt) signaling. The Wnt/β-catenin pathway, which is conserved, plays a crucial role in regulating stem cell pluripotency during biological development and determining the outcome of cell differentiation [99]. The Wnt signaling pathway is classified into canonical and noncanonical pathways. Noncanonical Wnt signaling operates without involving β-catenin and is activated by Wnt ligands, which bind to a receptor complex consisting of frizzled, receptor tyrosine kinase-like orphan receptor 1/2, or receptor tyrosine kinase. The canonical pathway is based on the stability of the transcription cofactor, β-catenin [100]. Wnt signaling is activated when Wnt ligands bind to frizzled receptors along with lipoprotein receptor-related protein 5/6 (LRP5/6) [101]. This binding induces the formation of multiprotein complexes (Dishevelled [Dvl in mammals, Dsh in *Drosophila*], CK1γ, Axin1, and GSK3β) and inhibits the disruption of complex activity. Consequently, β-catenin is stabilized and translocated to the nucleus, where it activates the transcription of Wnt target genes in collaboration with T-cell factor/lymphoid enhancer factor (TCF/LEF) [102]. This demonstrates that Chibby competes with β-catenin for TCF/LEF binding, consequently repressing catenin-mediated gene activation. Affinity-based purification/MS has identified 14–3-3ε and 14–3-3ζ as Chibby-binding partners. Chibby and 14–3-3 then form a complex with β-catenin, causing β-catenin to divide into the cytoplasm, antagonizing β-catenin signaling [103]. Castañeda et al. found that the 14–3-3 family members are widely involved in the regulation of PI3K/Akt/β-catenin signaling; 14–3-3ε enhances Akt/ε-catenin signaling by inhibiting PI3K and PDK1 [104]. Kosaka et al. reported that 14–3-3ε regulated the cardiomyocyte cycle through p27kip1 and played an important role in the proliferative development of dense ventricular myocardium [105]. A schematic of this process is shown in Fig. 2.

#### 14–3-3ε exerts anti-inflammatory, antiviral and anti-apoptotic effects through the tumor necrosis factor (TNF)α signaling pathway

TNFα is a pro-inflammatory cytokine that plays diverse roles in many physiological and pathological processes and is widely involved in the development of inflammatory diseases, viral infections, and cardiovascular diseases [106, 107]. TNFα activates the Ikappa-B (IκB) kinase (IKK)/NF-κB and mitogen-activated protein kinase (MAPK) pathways, which play a crucial role in the expression of pro-inflammatory cytokines and the initiation of various biological events such as apoptosis and necrosis [108, 109]. TNFα also exerts its biological effects by binding to cell surface receptors, namely, TNF receptor 1 (TNFR1) and TNFR2 [110]. TNFR1 sequentially recruits TNFR-associated death domain protein, TNFR-associated factor 2 (TRAF2), IKK, and the receptor-interacting protein, thereby activating NF-κB. Meanwhile, TNFR2 recruits TRAF1 and TRAF2 to induce similar downstream effects [111]. 14–3-3ε actively participates in the TNFα/NF-κB pathway network. Stimulation with TNFα results in increased interaction between 14–3-3ε and important components of the MAPK signaling module, which are located upstream of NF-κB. These components include transforming growth factor-β-activated kinase-1 (TAK1) and PPM1B [112]. As a signaling molecule upstream of the pathway, TAK1 promotes NF-κB-dependent transcription by directly phosphorylating IKK at Ser 177 and Ser 181 located within the IKKβ activation loop [113]. In addition, PPM1B plays an important role in terminating TNFα-mediated NF-κB activation by inactivating IKKβ [114].

**Fig. 3:**
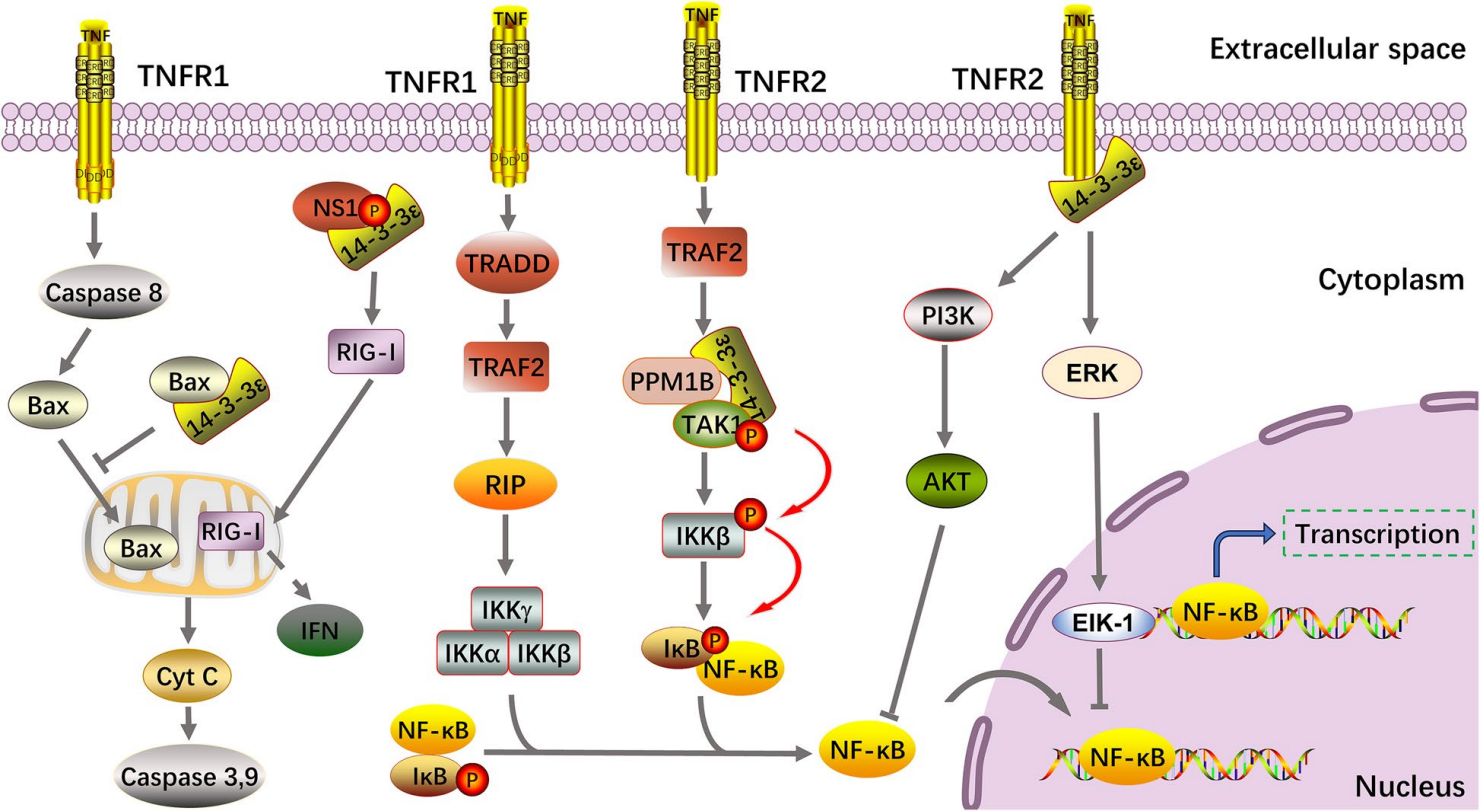
14–3-3ε exerts anti-inflammatory, antiviral, and anti-apoptotic effects via the TNF-ε signaling pathway. In response to TNF-α stimulation, 14–3-3ε forms a complex with TAK1 and PPM1B, and TAK1 phosphorylates IKKβ, ultimately promoting the entry of NF-κB into the nucleus. The interaction between Bax and 14–3-3ε inhibits the translocation of Bax to the mitochondria, ultimately inhibiting apoptosis. Additionally, the TNFR2/14–3-3ε complex promotes NF-κB activation and entry into the nucleus via PI3K/AKT and ERK/EIK-1. TNF, tumor necrosis factor; TAK1, transforming growth factor-β-activated kinase-1; IKK, Ikappa-B kinase

This pathway is associated with various diseases. Recent studies have demonstrated that 14–3-3ε is a constituent of the TNFR2 complex. Moreover, the TNFR2/14–3-3ε complex promotes an anti-inflammatory phenotype in macrophages affected by inflammatory arthritis [115, 116], thereby being a potentially novel target for the clinical management of rheumatoid arthritis and osteoarthritis. Priam et al. also demonstrated that 14–3-3ε binds directly to CD13 to trigger an inflammatory response, inducing a catabolic phenotype similar to that observed in osteoarthritis [117]. During viral infection, the threonine at amino acid 49 of influenza virus multifunctional NS1 interacts with 14–3-3ε [118, 119]. This interaction activates IFN expression by translocating RIG-I to the mitochondria, resulting in an antiviral effect. Additionally, the hepatitis virus directly binds to Bax and disrupts the interaction between Bax and 14–3-3ε. This disruption enhances the translocation of Bax to the mitochondria, leading to the release of Cyt c and activation of caspase-9 and caspase-3, ultimately inducing apoptosis [120, 121]. A schematic of this process is shown in Fig. 3.

#### 14–3-3ε competitively binds with glioma-associated oncogene homolog 1 (Gli) to inhibit Hh signaling

**Fig. 4:**
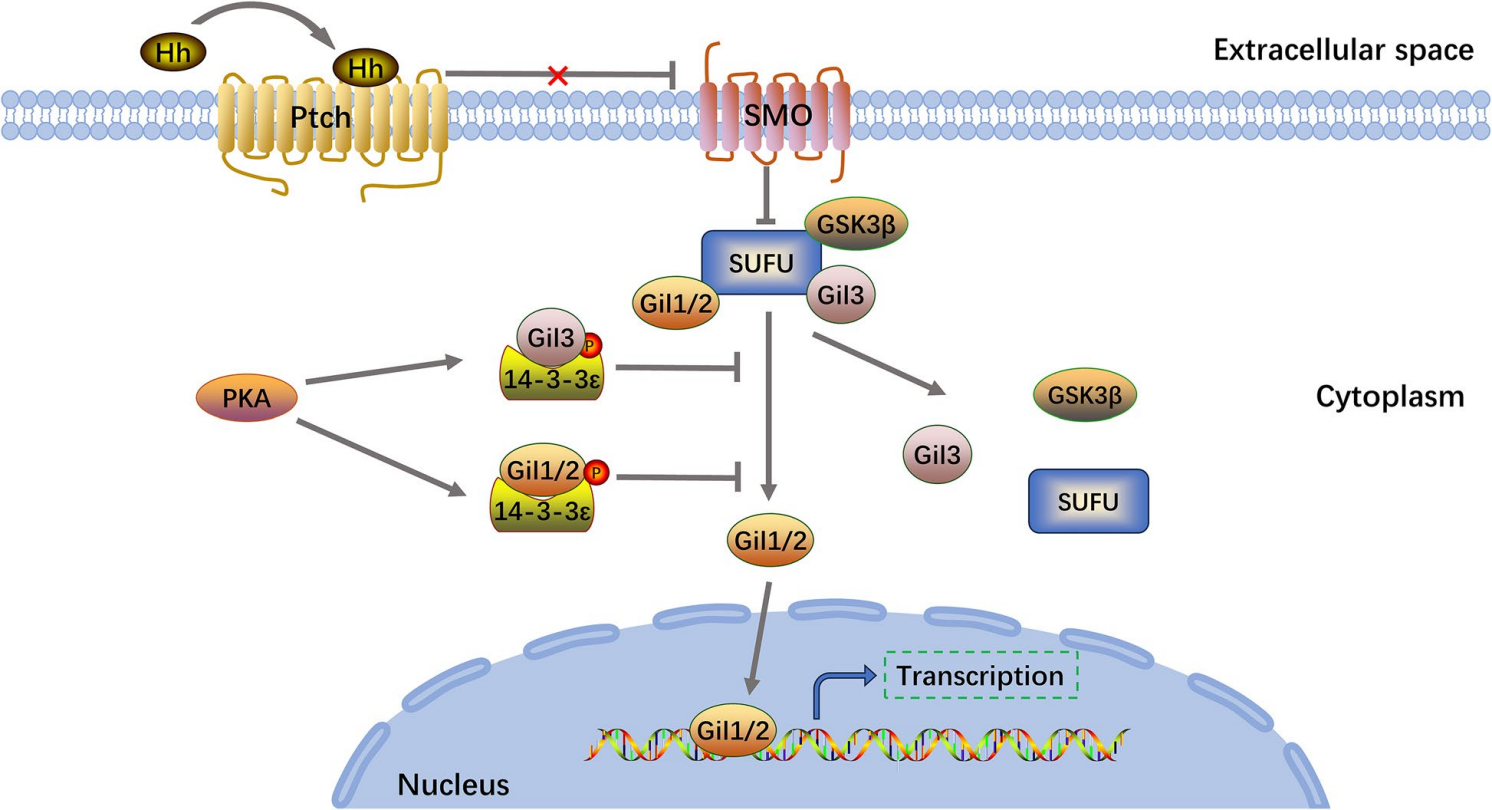
14–3-3ε competitively binds to Gli to inhibit the Hedgehog signaling pathway. PKA promotes the interaction of Gli with 14–3-3ε, inhibits Gli translocation to the nucleus, and ultimately suppresses transcription of downstream genes. Gli, glioma-associated oncogene homolog 1; PKA, protein kinase A

The Hh family of secretory proteins plays a key role in embryonic development and adult tissue homeostasis in many species, ranging from insects to mammals [122, 123]. The Hh signaling pathway in mammals primarily comprises Hh ligands, transmembrane proteins, nuclear transcription factors, and downstream target genes. Binding of the Hh ligand to the extracellular domain of Patched inhibits the receptor and eliminates its inhibitory effect on smoothened (Smo). As a result, Smo inhibits the sequestration of Sufu and the phosphorylation of PKA, CK1, and glycogen synthase kinase 3β, thereby preventing the proteolytic cleavage of Gli. Gli is translocated to the nucleus and promotes the transcription of downstream genes [124, 125]. Gli is also a transcriptional effector found at the end of the Hh signaling pathway. The vertebrate Gli gene family comprises three members: Gli1, Gli2, and Gli3. PKA phosphorylates Gli1, Gli2, and Gli3, and these proteins interact with 14–3-3ε through homologous sites. Further, PKA promotes the interaction between Gli and 14–3-3ε, as well as proteolysis, leading to the downregulation of Hh signaling [126]. A schematic of this process is shown in Fig. 4.

#### 14–3-3ε promotes cell proliferation through the Hippo-YAP signaling pathway

**Fig. 5:**
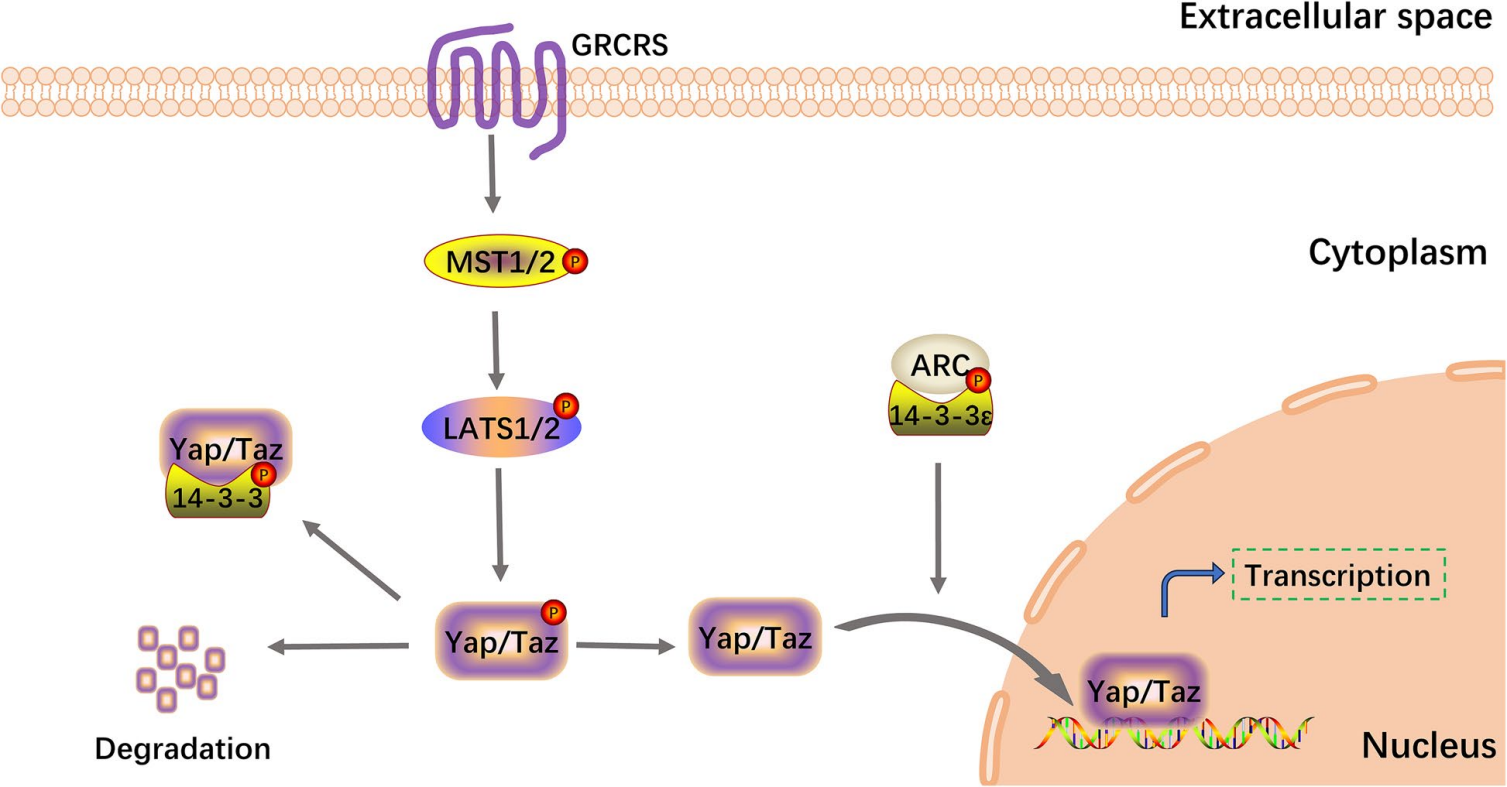
14–3-3ε promotes cell proliferation through Hippo-YAP signaling. ARC enhances nuclear localization of YAP/Taz by interacting with 14–3-3ε, ultimately promoting cell proliferation. YAP, Yes-associated protein; ARC, apoptosis repressor with a caspase recruitment domain; TAZ, transcriptional coactivator with PDZ-binding motif

The Hippo-YAP pathway regulates tissue growth in both the physiological and pathological states [127–130]. When the Hippo-YAP pathway is activated, phosphorylated Mammalian sterile-20-like1/2 promotes the phosphorylation of LATS1/2, resulting in the degradation of YAP and its transcriptional coactivator with PDZ-binding motif (TAZ). When the Hippo-YAP pathway is inhibited, YAP/TAZ undergoes dephosphorylation and translocates to the nucleus, thereby facilitating the transcription of downstream genes. The Hippo-YAP pathway plays an important role in cardiovascular disease [131], which is the leading cause of death worldwide and severely burdens society [132]. Vascular smooth muscle cells (VSMCs) are the main cell type in vessel walls and are involved in atherosclerosis [133]. During vascular injury, VSMCs can switch from a contractile to a proliferative phenotype, thereby promoting neointima formation [134]. Liu et al. demonstrated that ARC enhanced the nuclear localization of YAP through its interaction with 14–3-3ε and that ARC promoted cell proliferation and phenotypic regulation through 14–3-3ε/YAP signaling in VSMCs [27]. These findings provide new insights into the prevention and treatment of atherosclerosis. A schematic of this process is shown in Fig. 5.

### 14–3-3ε participates in tumorigenesis and development

17p13.3 is one of the chromosomal regions most frequently affected by allelic deletions in various human tumors [135–138], suggesting that 14–3-3ε located in this region is important in tumor development. 14–3-3ε is abnormally expressed in kidney tumors [139], colorectal cancer [140], melanoma [141], breast cancer [142], meningioma [143], ovarian cancer [144], and many other tumors and profoundly affects the process of tumor development. This demonstrates that 14–3-3ε plays a crucial role in cancer regulation and can serve as a potential clinical target. Current literature supports that 14–3-3ε is involved in the proliferation, apoptosis, migration, invasion, and resistance to chemoradiotherapy of various cancers. This process involves the interaction of 14–3-3ε with the proto-oncogene or oncogene product.

#### 14–3-3ε promotes cancer cell proliferation and inhibits cancer cell apoptosis

Uncontrolled cell proliferation is a key step in the development of cancer. Abnormalities in the Wnt/β-catenin [145] and ERK/MAPK [146–148] pathways contribute to uncontrolled cell proliferation. 14–3-3ε enhances hepatocellular carcinoma (HCC) cell proliferation and tumor growth by promoting the expression and nuclear translocation of β-catenin [149]. Raf-1 kinase inhibitory protein (RKIP) exerts its antitumor effects by inhibiting the Raf-regulated ERK/MAPK pathway [150]. Yan et al. found that RKIP and 14–3-3ε colocalize in cells and that 14–3-3ε played a tumor-promoting role by increasing phosphorylated RKIP and ERK levels and activating the ERK/MAPK pathway [151].

Cell cycle proteins play a role in tumor progression by inducing cell proliferation and apoptosis. CDC25 phosphatase plays a key role in the cell cycle by activating cell cycle protein-dependent kinases for mitosis and is considered a potential human oncogene [152]. Previous studies have demonstrated that CDC25A interacts with 14–3-3ε to regulate its subcellular localization, and it has been found to play a role in tumorigenesis [153]. In cutaneous squamous cell carcinoma (SCC), 14–3-3ε bound to CDC25A residue phosphoserines 178 and 507, promoting Akt/Bad/Survivin pro-survival signaling to suppress apoptosis [154]. In HeLa cells, 14–3-3ε impeded cell cycle progression by blocking CDC25A degradation [155]. In addition, CDC25B is regarded as a potential oncogene highly expressed in tumors [156, 157]. Another study demonstrated that 14–3-3ε inhibited gastric cancer (GC) cell proliferation by reducing MYC and CDC25B expression [158]. Additionally, 14–3-3ε knockdown inhibited the proliferation of GC cells by regulating cell cycle proteins, specifically by downregulating cyclin E and upregulating p27^kip1^ [159].

14–3-3ε is regulated by non-coding RNAs in malignant tumors. miR-31-5p acts as a tumor suppressor in human prostate cancer cells. miR-31-5p targets 14–3-3ε via the PI3K/AKT/Bcl-2 signaling pathway, thereby inhibiting prostate cancer cell proliferation and promoting apoptosis. This provides an experimental and theoretical basis for its use as a potential prognostic biomarker and therapeutic target for prostate cancer [160]. In addition, the transcription of the non-coding RNA LINC00920 is activated by ERG, which in turn promotes prostate cancer cell survival via the 14–3-3ε-FOXO pathway [161].

14–3-3ε also regulates tumor cell proliferation and apoptosis through self-modification or interactions with other proteins. The acetylation of 14–3-3ε at K50 significantly reduced cholangiocarcinoma cell proliferation in vitro and inhibited tumor xenograft growth in vivo [162]. FOXO3a transcriptional activity is deactivated through Akt-mediated phosphorylation of serine 253 (S253), resulting in the binding of FOXO3a to 14–3-3ε. The interaction between phosphorylated FOXO3a and 14–3-3ε facilitates the exclusion of FOXO3a from the nucleus, which ultimately leads to the proliferation of cancer cells [163]. Low metallothionein-1 (MT-1) expression is often predictive of a poor prognosis in HCC [164]. 14–3-3ε suppresses MT-1 expression by inducing ZNF479 expression, and MT-1 overexpression counteracts the cell proliferation and tumor growth induced by 14–3-3ε [164]. LNK is a widely studied adapter protein that is found in both normal and malignant hematopoietic cells. LNK overexpression has been recently found to result in increased proliferation and anti-apoptotic signaling by activating the Akt-NFκB-Bcl-2/Bcl-xL pathway, and its oncogenic effect largely depends on 14–3-3ε/γ [165].

#### 14–3-3ε promotes the invasion and metastasis of cancer cells

14–3-3ε is extensively involved in tumor invasion, migration, and metastasis through a sophisticated regulatory network. 14–3-3ε overexpression increased the risk of HCC metastasis by 4.6-fold [166]. Gong et al. also reported that the expression level of 14–3-3ε was negatively associated with the differentiation of GC tissue, while it was positively associated with tumor invasion depth, lymph node metastasis, lymph node invasion, and tumor metastasis stage [159]. In addition, MS and immunohistochemistry analysis of 14–3-3ε in primary and metastatic melanoma tissues revealed that 14–3-3ε levels were significantly elevated in aggressive melanoma tissues [167]. In laryngeal SCC (LSCC) tissues, 14–3-3ε promoted apoptosis and inhibited the invasiveness of LSCC [168]. Collectively, these results support that 14–3-3ε is a potential therapeutic target in tumor metastasis.

Epithelial–mesenchymal transition (EMT) is a developmental process in which epithelial cells lose apical-basal cell polarity and eventually transform into invasive mesenchymal cells, which play a key role in early tumor embryogenesis and transformation to invasive malignancy. EMT is characterized by the loss of adhesion, loss of epithelial markers (E-cadherin, claudin, and occludin), and gain of mesenchymal markers (N-cadherin and vimentin) [169]. A study reported that 14–3-3ε acted as an important regulator of HCC cell metastasis by promoting EMT through the regulation of Zeb-1/E-calmodulin expression [170]. Another study conducted by the same researchers showed that 14–3-3ε could also hinder the expression of aldoketone reductase family 1 member B10 (AKR1B10) by activating β-catenin signaling. Low levels of AKR1B10 expression significantly increase the EMT in advanced HCC [149].

Focal adhesion kinase (FAK) is associated with the survival, proliferation, and migration of cancer cells. FAK overexpression is significantly associated with an increased risk of extrahepatic metastasis. An in-depth study demonstrated that 14–3-3ε promoted the activation of NF-κB and facilitated its nuclear translocation. In addition, NF-κB bound to the FAK promoter region, which promoted the metastasis of HCC tumors [171]. Increased Par-3 expression is associated with distant metastasis and low overall survival in patients with HCC [170], and 14–3-3 interacts with Par-3 to control cell polarity [172]. It can be hypothesized that 14–3-3ε synergistically controls cell polarity along with Par-3, thereby promoting invasive migration of HCC cells. Breast cancer metastasis suppressor 1 (BRMS1) inhibits metastasis of multiple tumor types without blocking tumorigenesis [173]. BRMS1 was reported to be post-translationally regulated by casein kinase 2 catalytic subunit phosphorylation of nuclear BRMS1 on serine 30, leading to its export from the nucleus for degradation through 14–3-3ε mediation, which ultimately resulted in lung cancer metastasis [142]. 14–3-3ε expression is upregulated in esophageal cancer and is positively correlated with metastasis and poor tumor prognosis [174]. Nuclear factor 45 promotes cell invasion in esophageal SCC (ESCC) cell lines by upregulating Rac1/Tiam1 signaling through the 14–3-3ε protein [175]. Notably, although 14–3-3ε has been confirmed to promote the migration of cancer cells, one study also reported that 14–3-3ε inhibited this process [176]. HSP27 phosphorylation plays a crucial role in regulating F-actin polymerization, actin cytoskeleton organization, and the resulting dynamics of actin filaments, ultimately promoting cell migration. However, in HeLa cells, the interaction between 14–3-3ε and MAPK-activated protein kinase 5 hinders this process by inhibiting HSP27 [176]. In conclusion, 14–3-3 regulates a complex signaling network that participates in the invasion, migration, and metastasis of cancer cells.

#### Role of 14–3-3ε in chemoradiotherapy resistance

14–3-3ε is involved in resistance to many chemotherapeutic agents and performs different chemoradiotherapy resistance functions. In CML-BC-resistant cells, 14–3-3ε increases p38-MAPK activity, leading to imatinib resistance [177]. In addition, 14–3-3ε might affect the actin and microtubulin cytoskeletal systems in cancer cells, thereby contributing to resistance to vinca alkaloid [178]. Another study confirmed that 14–3-3ε negatively affected the ability of the chemotherapeutic agent etoposide to trap topo II in cleavable complexes with DNA, thereby preventing DNA strand breaks [179]. Additionally, high pretreatment levels of 14–3-3ε predicted poor prognosis and chemoresistance in asparaginase therapy [180]. However, positive 14–3-3ε expression led to proteasome inhibitor sensitivity in multiple myeloma [181]. Bleomycin resistance was inhibited by phosphorylation-dependent binding between 14–3-3ε and major vault protein [182]. Collectively, these findings support that the role of 14–3-3ε in drug resistance differs by cancer type, but it can be worthwhile to affirm that 14–3-3ε is closely associated with drug resistance in multiple tumor types.

### Potential clinical significance of 14–3-3ε

The diverse functions of 14–3-3ε are influenced by numerous chaperones, which play a crucial role in different disease processes. As a 14–3-3ε stabilizer, fusicoccin-A (FC-A) can further exert beneficial effects based on the physiological functions of 14–3-3ε. In tumor treatment, chemotherapy resistance is a significant challenge that greatly affects tumor prognosis. Studies have confirmed the involvement of 14–3-3ε in various chemoresistance processes. Several drugs that target 14–3-3ε have been discovered, and they demonstrate various effects such as anti-tumor, anti-atherosclerosis, anti-oxidative aging, and anti-depression effects.

#### Drugs targeting 14–3-3ε

Various drugs, mainly antitumor drugs, have been discovered to target 14–3-3. In cholangiocarcinoma cells, cisplatin not only plays a therapeutic role, but also increases the expression of 14–3-3ε, thereby forming a survival mechanism by activating the PI3K/AKT pathway. Treatment with low-concentration arsenic trioxide improves the chemotherapeutic efficiency of cisplatin in cholangiocarcinoma cells by inhibiting the survival mechanism mediated by 14–3-3ε [183]. PPARδ agonists have been used in the treatment of dyslipidemia and metabolic syndrome and are promising agents for the treatment of several metabolic disorders [184]. Curcumin is a natural plant polyphenol extracted from turmeric rhizomes. Curcumin induces apoptosis in MG-63 cells [185] and HT-29 cells [186] by targeting 14–3-3ε. In addition, proteasome inhibitors, a novel anticancer drug, cause cytotoxicity in glioma cells through the downregulation of 14–3-3ε and 14–3-3θ/τ and the activation of apoptosis signal-regulating kinase 1 [187]. 4-Amino-2-trifluoromethyl-phenyl retinate reduces the binding of 14–3-3ε to filaggrin A, thereby inducing the nuclear localization of filaggrin A and ultimately inducing G0/G1 phase arrest [188]. Xu et al. demonstrated that betulinic acid induces apoptosis in HeLa cells by downregulating 14–3-3β and 14–3-3ε levels [189].

PPARδ upregulates 14–3-3ε in human endothelial cells via CCAAT/ EBPβ, which is beneficial in atherosclerosis by resisting apoptosis [190]. Ganoderic acid D is a *Ganoderma lucidum*-derived triterpenoid. Recent studies have shown that Ganoderic acid D prevents oxidative stress-induced senescence by targeting 14–3-3ε to activate the CaM/CaMKII/NRF2 signaling pathway in mesenchymal stem cells [191]. Fluoxetine, a drug commonly used for depression and anxiety, upregulates the expression of 14–3-3ε [192]. Rosiglitazone induces the binding of PPARγ to 14–3-3ε, thereby increasing its transcription of 14–3-3ε [193]. Ultimately, upregulation of 14–3-3ε inhibits apoptosis [194]. In conclusion, 14–3-3ε binds to a large number of proteins and acts as a molecular chaperone, providing new concepts for the development of related drugs.

#### Novel 14–3-3ε stabilizer-FC-A

Currently, the more established 14–3-3 targeting drug molecule is FC-A. The small molecule FC-A, which is produced by *Phomopsis amygdali*, stabilizes the interactions between 14–3-3 and client proteins. FC-A and its derivatives have been found to play positive roles in the treatment of cystic fibrosis and cancer [195, 196]. Based on the anti-apoptosis role played by 14–3-3ε and the key role played by the ventrolateral orbital cortex (VLO) in depression [197, 198], Zhao et al. evaluated the effects of the 14–3-3ε stabilizer FC-A on mice with major depressive disorder. Their results confirmed that FC-A treatment promoted 14–3-3ε expression in VLO, prevented neuronal apoptotic signaling in VLO by inhibiting Bad-mediated apoptosis, and alleviated depressive-like behavior after chronic unpredictable mild stress [199]. However, we could not find other studies on FC-A regulation by controlling 14–3-3ε levels in other diseases, indicating a significant research gap. Further studies are required to fully understand the functions of FC-A and its derivatives.

#### 14–3-3ε as a biokine favoring osteogenesis

Adipose-derived mesenchymal stem cells (ASCs) are a promising treatment modality for bone tissue regeneration. A new type of nanofibrous biomimetic matrix composed of poly (ε-caprolactone), nanometric hydroxyapatite particles, and 14–3-3ε protein enhances the osteogenicity of ASCs [200, 201]. Three-dimensional printing can be used to assemble cells or replicate natural tissues in a spatially controlled manner, and biopolymer-based hydrogels are primarily used in this process. Owing to their high-water content and physical properties, hydrogels can mimic the extracellular matrix. The 14–3-3ε protein has positive effects on cell adhesion and proliferation, and purified human 14–3-3ε recombinant protein-loaded GelMA/AlgH hydrogel significantly improves the osteogenic differentiation of human adipose-derived stem cells [202].

#### TAT-14–3-3ε fusion protein against cerebral ischemia/reperfusion injury

Human immunodeficiency virus-1 transcription activator is an attractive drug delivery tool that enters cells in a nontoxic and efficient manner. It is also capable of carrying a wide range of biologically active substances of different sizes and properties into cells, such as proteins, peptides, small-molecule compounds, and dyes [203]. Based on the great potential of 14–3-3ε to inhibit apoptosis [28], Zhu et al. prepared a transcription activator (TAT)-14–3-3ε fusion protein and used it to perform preliminary experiments in transient focal cerebral ischemia model rats. Their results showed that pre- or post-ischemic treatment with TAT-14–3-3ε significantly increased the number of surviving neurons and attenuated neuronal apoptosis [204]. This fusion protein better compensated for the inability of large-molecular-weight substances, such as 14–3-3, to cross the blood–brain barrier.

#### 14–3-3ε levels act as a predictive factor and prognostic factor in neurodegenerative diseases

17p13.3 microduplication syndrome is a genetic syndrome characterized by duplications of various sizes in the 17p13.3 chromosome locus, mainly involving the *LIS1* and/or *YWHAE* genes [205]. Chromosome 17 instability is linked to the development of various diseases such as morphological brain disorders, mental illnesses, epilepsy, and tumors [206–209]. The analysis of sera from adult comatose survivors of ventricular fibrillation or pulseless ventricular tachycardia indicated that 14–3-3ε was a serum-specific biomarker of poor neurological recovery [210]. The *YWHAE* gene, which encodes the 14–3-3ε protein, is considered a susceptibility gene for schizophrenia [211]. For instance, in a study conducted in a Japanese population, *YWHAE* was found to be associated with schizophrenia [211]. 14–3-3ε is also a potential suicide susceptibility gene [212].

## Discussion

**Fig. 6:**
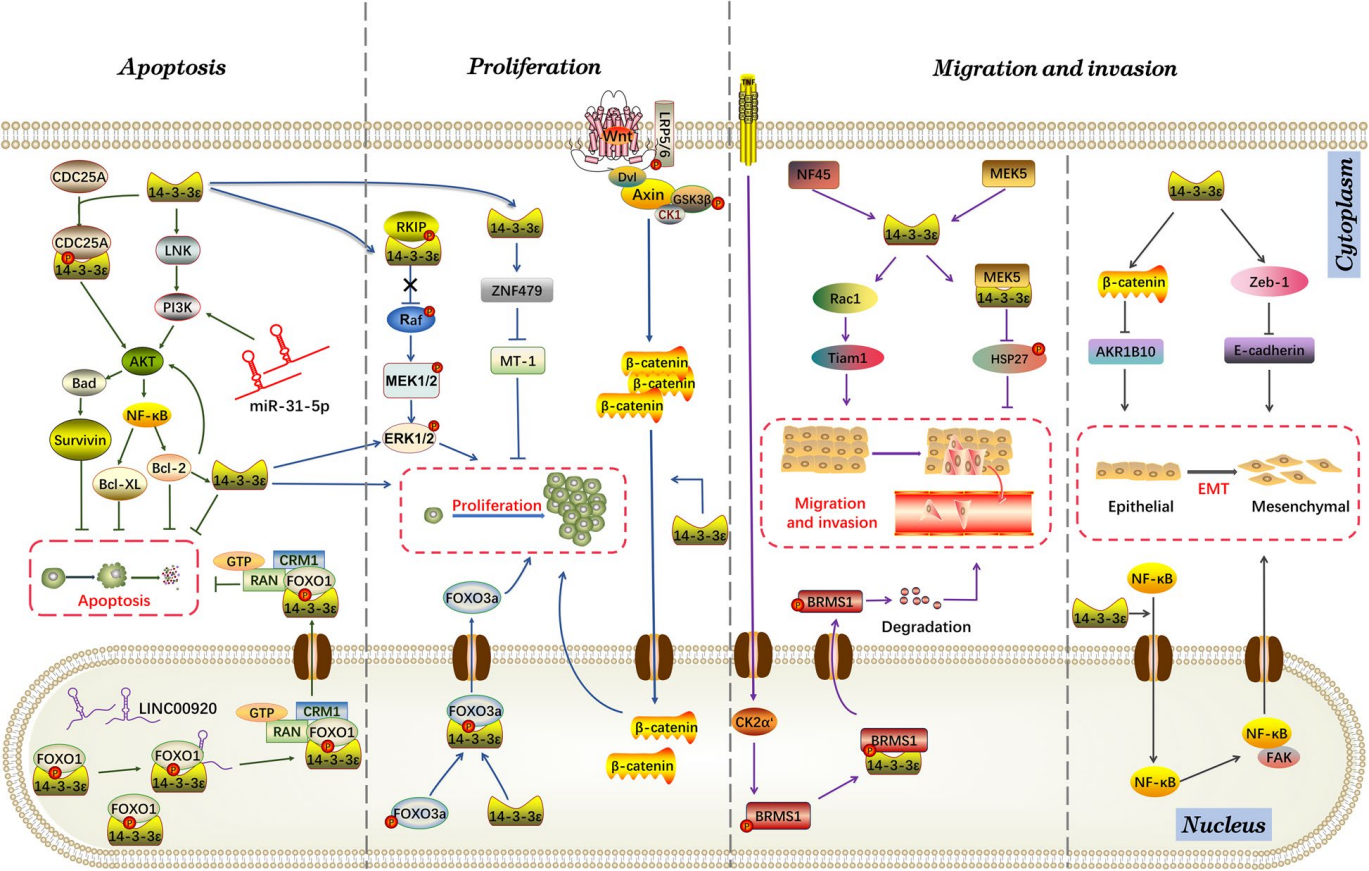
14–3-3ε’s specific mechanisms involved in tumor developmen

14–3-3 proteins belong to a family of multifunctional proteins that are highly conserved across different species. 14–3-3 proteins can exist as homodimers and heterodimers, with a dynamic equilibrium between dimers and monomers in vivo. This balance can be affected by phosphorylation or other posttranslational modifications, as well as by interactions with small molecules [213]. The unique structure of the 14–3-3 dimer makes it a novel biological tool for regulating cell signaling pathways and gene expression in cancer cells. Previous research has shown that 14–3-3 proteins are ordered hubs that can interact with disordered phosphorylation targets. This interaction can be used to create a specific binding pocket for the two proteins with the help of a small-molecule stabilizer [213]. In summary, 14–3-3 proteins offer a promising avenue for discovering new biological tools that can be utilized to investigate gene expression and cancer signaling pathways (Fig. 6) and gain insights into neurodegenerative diseases.

To date, over 1,200 protein partners, including protein kinases, phosphatases, receptors, and transcription factors, have been shown to interact with 14–3-3 proteins [14, 15]. These interactions are associated with the pathogenesis of various diseases. Concurrently, 14–3-3 proteins are involved in the regulation of multiple signaling pathways, including intracellular signal transduction, cell cycle regulation, apoptosis, proliferation, and gene expression. FC-A serves as a crucial foundation for this type of therapy and can guide the development of targeted small molecules. Thus, 14–3-3 proteins are a potential target for pharmaceutical intervention. Moreover, there is the potential for drug molecules to specifically interfere with the key signal transduction pathways involved in disease states. The role of 14–3-3ε in tumor therapy has gained significant attention. Owing to its molecular chaperone properties, modulating the interaction of 14–3-3ε with oncogenes or transcriptional regulators may be a potential strategy for tumor therapy. The regulatory mechanism of 14–3-3ε in tumor drug resistance is complex and thus requires further research. However, 14–3-3ε can serve as a potential target to overcome tumor drug resistance. For the treatment of osteoarthritis and cerebral ischemia/reperfusion injury, the integration of new technologies can support the effectiveness of 14–3-3 and offer new perspectives for the development of related drugs.

Significant research has been dedicated to 14–3-3 dimers, but there is a lack of research on 14–3-3 monomers. Despite the high homology of the 14–3-3 family of proteins, each subunit has unique characteristics. 14–3-3ε plays an irreplaceable role in cell proliferation, apoptosis, autophagy, cell cycle regulation, repolarization of cardiac action, cardiac development, intracellular electrolyte homeostasis, neurodevelopment, and innate immunity. 14–3-3ε has been implicated in the pathogenesis of various diseases including Parkinson’s, cerebral ischemia–reperfusion, stroke, epilepsy, Miller-Dieker syndrome, and Alzheimer’s. More worthy of our attention is the important role of 14–3-3ε in cancer, as it can be used as a poor prognosis marker for liver cancer, colorectal cancer, pediatric astrocytoma, and ESCC. 14–3-3ε promotes cancer cell invasion and migration mainly by regulating cancer cell apoptosis, proliferation, and EMT. However, the significance of 14–3-3ε expression in GC remains unclear and requires further investigation in larger clinical trials to fully understand its effects. The abnormal expression of 14–3-3ε in various cancers makes it an independent prognostic marker, but its abnormal expression in specific tumor types requires further study. Clinical drug research can be conducted on the abnormal expression of 14–3-3ε in tumors. Targeted therapy can then be developed based on the 14–3-3ε mechanism pathway, with the ultimate goal of improving cancer prognosis.

## Conclusion

In conclusion, despite the considerable advancement, there remain several unexplored aspects pertaining to our comprehension of 14–3-3ε. The distinctive structure and extensive range of binding partners of 14–3-3 proteins present opportunities for their involvement in clinical diagnosis and treatment. Furthermore, recognizing the significance of 14–3-3ε in cancer treatment is crucial, as its role in drug resistance, proliferation, and migration holds great potential for the development of novel drugs.

## Data Availability

No available.

## Abbreviations

AKR1B10: Aldoketone reductase family 1 member B10
Apaf-1: Apoptotic protease activating factor-1
ARC: Apoptosis repressor with caspase recruitment domain
ASCs: Adipose-derived mesenchymal stem cells
BRMS1: Breast cancer metastasis suppressor 1
CDC25: Cell division cyclin 25
CDK1: Cyclin-dependent kinase 1
CLIC4: Chloride intracellular channel 4
Cyt c: Cytochrome c
EIF2α: Eukaryotic initiation factor-2α
EMT: Epithelial–mesenchymal transition
ESCC: Esophageal squamous cell carcinoma
SCC: Squamous cell carcinoma
FAK: Focal adhesion kinase
FC-A: Fusicoccin-A
FoxO1: Forkhead box protein o1
GAPs: GTPase-activating proteins
GC: Gastric cancer
Gli: Glioma-associated oncogene homolog 1
HCC: Hepatocellular carcinoma
HERG: Human ether-a-go-go related gene
Hh: Hedgehog
IKK: IκB kinase
LRP5/6: Lipoprotein receptor‐related protein 5/6
LSCC: Laryngeal squamous cell carcinoma
MAPK: Mitogen-activated protein kinase
MAVS: Mitochondria antiviral signaling protein
MK5: MAPK-activated protein kinase 5
MT-1: Metallothionein-1
NADE: P75NTR-associated cell death executor
NCX: Na + /Ca2 + exchanger
Pins: Partner of Inscuteable
PKA: Protein kinase A
PMCA: Plasma membrane Ca2 + -ATPase
PPAR: Peroxisome proliferator-activated receptor
PPM1B: Protein phosphatase, Mg 2 + /Mn 2 + -dependent,1B
RIG-I: Retinoic acid-inducible gene I
RKIP: Raf-1 kinase inhibitory protein
SLP-76: SH2 domain-containing leukocyte protein of 76 kD
Smo: Smoothened
TAK1: Transforming growth factor-β-activated kinase-1
TAT: Transcription activator
TAZ: Transcriptional coactivator with PDZ-binding motif
TCF/LEF: T cell factor/lymphoid enhancer factor
TGFβ: Tumor growth factor β
TNFR1: Tumor necrosis factor receptor 1
TNFR2: Tumor necrosis factor receptor 2
TNFα: Tumour necrosis factor α
TRAF1: TNFR-associated factor 1
TRAF2: TNFR-associated factor 2
TβRI: Type I TGFβ receptors
TβRII: Type II TGFβ receptors
TβRIII: Type III TGFβ receptors
VLO: Ventrolateral orbital cortex
VSMCs: Vascular smooth muscle cells
Wnt: Wingless
YAP: Yes-associated protein
